# Paterson‐Brown Kelly Syndrome (also commonly known as Plummer‐Vinson Syndrome)

**DOI:** 10.1002/ccr3.3127

**Published:** 2020-07-20

**Authors:** Wilson Guo Wei Goh, Deborah Chieh Yih Ng, Jun Xuan Ng, Kheng Tian Lim

**Affiliations:** ^1^ General Surgery Khoo Teck Puat Hospital Singapore City Singapore; ^2^ General Surgery Khoo Teck Puat Hospital Singapore City Singapore; ^3^ Yong Loo Lin School of Medicine National University of Singapore Singapore City Singapore

**Keywords:** esophageal dysphagia, iron deficiency anemia, Paterson‐Brown‐Kelly Syndrome

## Abstract

The dysphagia in this condition is usually associated with iron deficiency anemia and esophageal webs. Iron supplementation and regular surveillance are required for monitoring of malignant transformation into esophageal squamous cell carcinoma.

## Graphical abstract

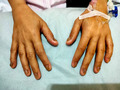

## 1 INTRODUCTION

**Figure 1:**
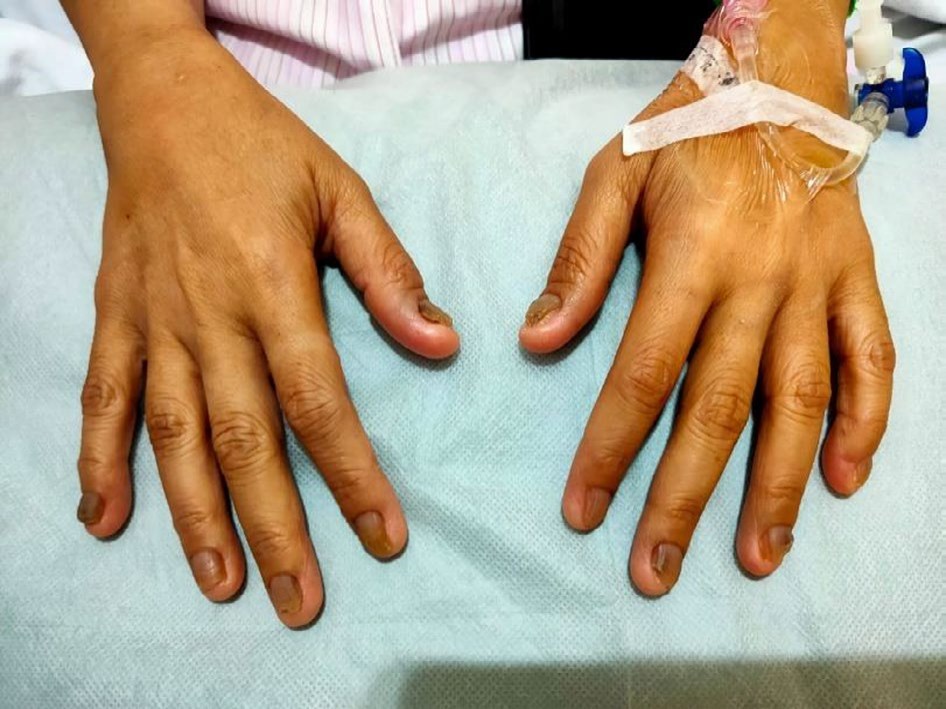
Koilonychia

**Figure 2:**
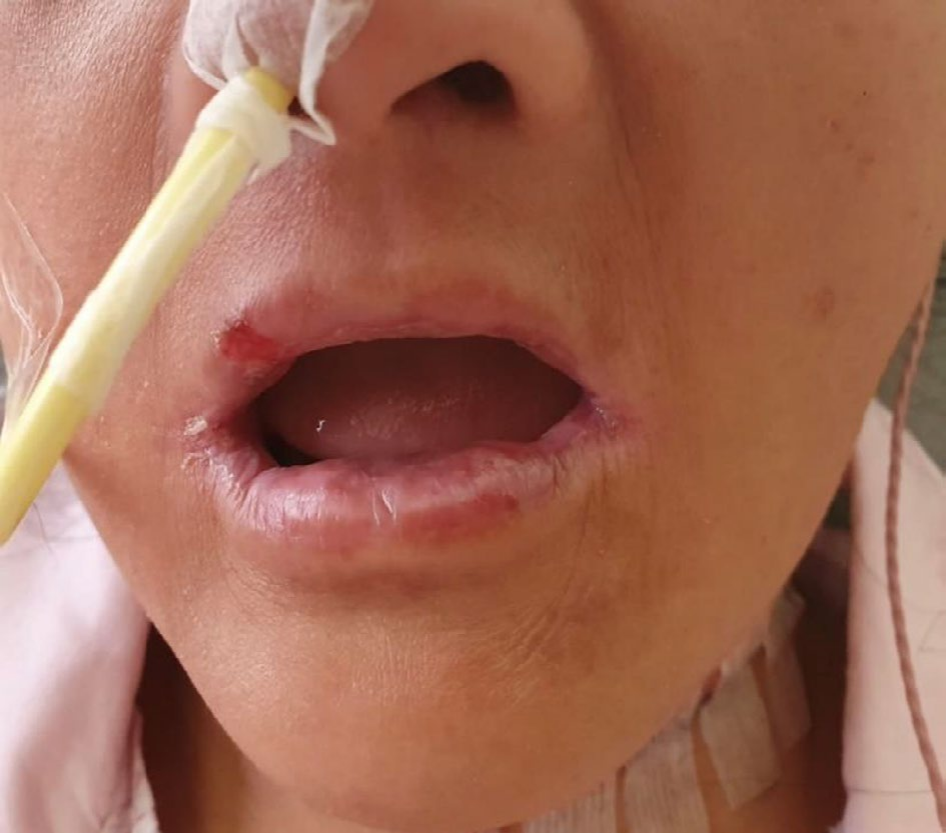
Angular stomatitis

**Figure 3:**
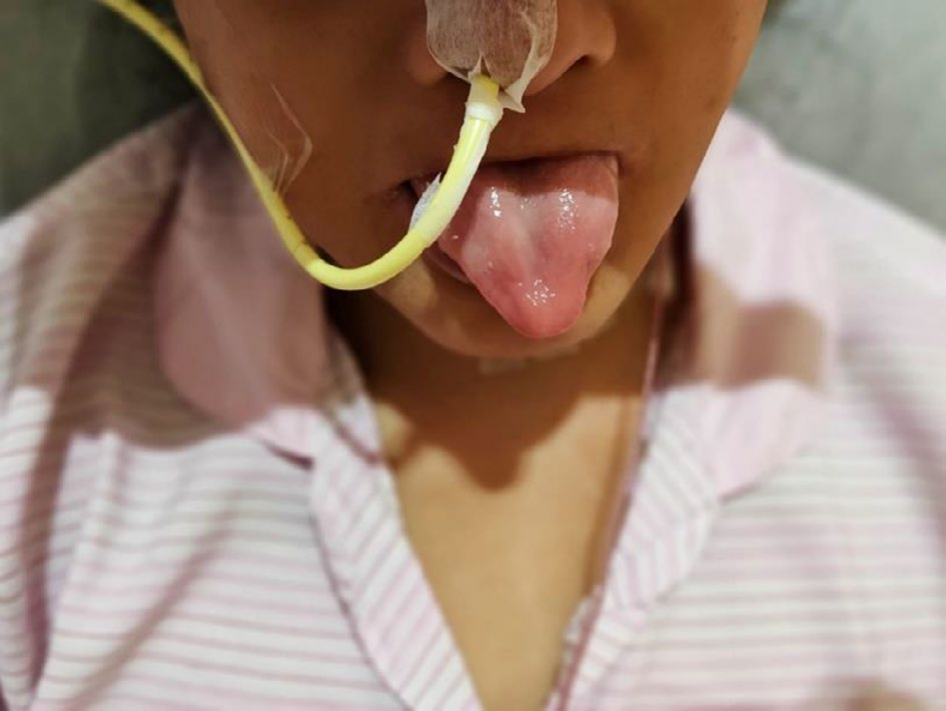
Glossitis—loss of tongue papillae

A 46 year‐old Malay female presents with symptoms of mechanical esophageal dysphagia. Physical examination revealed the following: (Figures 1, 2, 3).

## 2 WHAT IS THIS CONDITION?

Paterson‐Brown Kelly syndrome ^1^, ^2^ (also known as Plummer‐Vinson syndrome) comprises the clinical triad of dysphagia, esophageal webs, and iron deficiency anemia. This syndrome commonly affects females aged 40‐70 and is rare in the Asian context.

Symptoms include dysphagia, which is usually progressive, painless, and tends to involve solids initially. Patients may also present with symptoms of anemia or symptoms suggestive of aspiration pneumonia. Other clinical findings include koilonychia, angular stomatitis/cheilitis, and glossitis. Occasionally, this syndrome may be associated with Zenker's diverticulum, which is a differential diagnosis.

Upper endoscopy revealed esophageal strictures and blood investigations were suggestive of iron deficiency anemia. This corroborates with examination findings of iron deficiency anemia (Figures 1, 2, 3).

Balloon dilation of the strictures was performed, and she was simultaneously started on iron supplementation. Iron repletion therapy is associated with symptomatic improvement of dysphagia and slower malignant degeneration. She is planned for regular surveillance of malignant transformation of the hypopharynx or the upper esophagus with upper endoscopy.

## CONFLICT OF INTEREST

None declared.

## AUTHOR CONTRIBUTIONS

WGWG and JXN: were involved in writing this article. KTL and DCYN: were mentors in this process.
